# Current-Mode Shadow Filter with Single-Input Multiple-Output Using Current-Controlled Current Conveyors with Controlled Current Gain

**DOI:** 10.3390/s24020460

**Published:** 2024-01-11

**Authors:** Montree Kumngern, Fabian Khateb, Tomasz Kulej, Martin Kyselak, Somkiat Lerkvaranyu, Boonying Knobnob

**Affiliations:** 1 Department of Telecommunications Engineering, School of Engineering, King Mongkut’s Institute of Technology Ladkrabang, Bangkok 10520, Thailand; montree.ku@kmitl.ac.th (M.K.); somkiat.le@kmitl.ac.th (S.L.); 2 Department of Microelectronics, Brno University of Technology, Technická 10, 601 90 Brno, Czech Republic; 3 Faculty of Biomedical Engineering, Czech Technical University in Prague, Nám. Sítná 3105, 272 01 Kladno, Czech Republic; 4 Department of Electrical Engineering, Brno University of Defence, Kounicova 65, 662 10 Brno, Czech Republic; martin.kyselak@unob.cz; 5 Department of Electrical Engineering, Czestochowa University of Technology, 42-201 Czestochowa, Poland; kulej@el.pcz.czest.pl; 6 Faculty of Engineering, Rajamangala University of Technology Thanyaburi, Pathum Thani 12110, Thailand; kboonying@rmutt.ac.th

**Keywords:** shadow filter, current-mode filter, current-controlled current conveyor, second-generation current conveyor

## Abstract

In this paper, a novel current-mode shadow filter employing current-controlled current conveyors (CCCIIs) with controlled current gains is presented. The CCCII-based current-mode shadow filters are resistorless and can offer a number of advantages such as circuit simplicity and electronic tuning capability. The proposed shadow filters offer five filtering functions, i.e., low-pass, high-pass, band-pass, band-stop, and all-pass functions, in the same topology. Furthermore, no component matching condition is required to realize all the transfer functions. The natural frequency and quality factor adjustment is possible by using the CCCII current gains without the need to use external amplifiers, all capacitors are grounded, and the filter terminals offer low-input and high-output impedance. To verify the functionality and feasibility of the new topologies, the proposed circuits were simulated using SPICE and the transistor model process parameters NR100N (NPN) and PR100N (PNP) from AT&T’s bipolar arrays ALA400-CBIC-R. The simulation results are consistent with the theory. The CCCII experimental setup was designed using commercially available 2N3904 (NPN) and 2N3906 (PNP) transistors with a supply voltage of ±2.5 V. The measurement results confirm the performance of the designed filters.

## 1. Introduction

Over the last decade, second-generation current conveyors (CCIIs) have been used to realize current-mode analog circuits. This is because CCII-based circuits offer better signal bandwidth, higher linearity, circuit simplicity, and wider dynamic range performances compared with the operational amplifiers (op-amps)-based circuits [1,2]. In addition, a CCII is simpler to implement compared to the op-amp structure.

**Figure 1:**
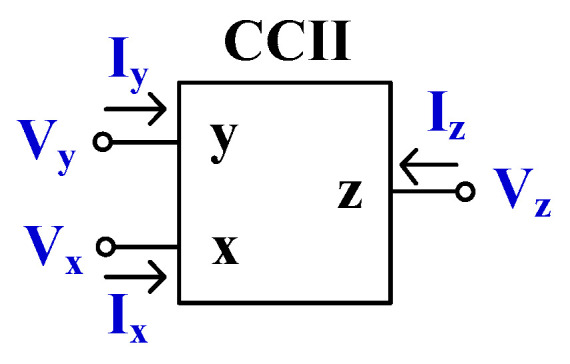
Electrical symbol of CCII.

Usually, a conventional CCII has three terminals (x-, y-, and z-terminal) [3]. Its electrical symbol is shown in Figure 1, while its terminal characteristics in ideal case are given by Equation (1).

(1) $$\left(\begin{array}{c} I_{y}\\ V_{x}\\ I_{z} \end{array}\right)=\left(\begin{array}{ccc} 0 & 0 & 0\\ 1 & 0 & 0\\ 0 & 1 & 0 \end{array}\right)\left(\begin{array}{c} V_{y}\\ I_{x}\\ V_{z} \end{array}\right)$$

It can be noted that the y-terminal is a voltage input that has a high impedance level (ideally infinity), the x-terminal is a voltage signal output and also a current signal input, with low impedance level (ideally zero), and the z-terminal is a current output with a high impedance level (ideally infinity). In practice, the parasitic resistance at the x-terminal (*R_x_*) of the CCII can be controlled by its bias current, which can be used as a design parameter. Such a device is called a current-controlled current conveyor (CCCII) [4]. Circuits based on CCCII can thus reduce the number of passive resistors and offer the possibility of electronic control.

It should be noted that in the ideal case is *V_x_* = *V_y_* for CCII and *V_x_* = *I_x_R_x_* + *V_y_* for CCCII, while *I_z_* = *I_x_* for both circuits. Note that the voltage and current gain in these formulas is equal to one. To increase the functionality, the CCCII with controlled current gain has also been proposed [5]. This device offers an adjustable current gain between the z- and x-terminals, which can be used as a design parameter for such applications as filters and oscillators. The CCII/CCCII can be realized using bipolar junction transistor (BJT) technology [5] or complementary metal oxide semiconductor (CMOS) technology [6,7]. In this work, a CCCII with controlled current gain is used to realize current-mode shadow filters.

The shadow filter was first introduced in [8]. The concept of the conventional shadow filter is to use an external amplifier to adjust the natural frequency and the quality factor of the second-order filters without changing the value of parameters such as capacitances and resistances of the original topology. However, the shadow filter in [8] does not provide independent control of the natural frequency and the quality factor. To obtain independent control of the above-mentioned parameters, the shadow filter was further developed, and two new systems were proposed [9]. The first system in [9] consists of a second-order filter and an amplifier. The LP and BP outputs are summed and amplified by the amplifier, and the output signal of the amplifier is summed with the input signal. Thus, the quality factor can be controlled by an external amplifier without changing the natural frequency. The second system in [9] was further developed by adding another amplifier to the first system. Thus, the second system in [9] consists of a second-order filter and two external amplifiers. The first amplifier is used to amplify the BP output, while the second one is used to amplify the LP output, and the output signals of the two amplifiers are summed with the input signal. Consequently, the quality factor can be modified by the first external amplifier, while the natural frequency can be modified by the second one. In this work, the two systems of shadow filters in [9] will be designed using CCCII with controlled current gain as active elements. It will be shown that the function of external amplifiers can be obtained using the current gains of CCCIIs.

Many shadow filters (also known as frequency-agile filters) have been introduced [10,11,12,13,14,15,16,17,18,19,20,21,22,23,24,25,26,27,28,29,30,31,32,33,34]. These filters can be used for various radio applications and sensor networks including environmental monitoring, vital signs monitoring, and military applications. Considering the operating mode of these filters, they can be divided into three operating modes: voltage-mode [10,11,12,13,14,15,16,17,18,19,20,21,22], current-mode [23,24,25,26,27,28,29,30,31], and mixed-mode (or multi-mode) [32,33,34].

Considering the active devices used to realize the voltage-mode shadow filters in [10,11,12,13,14,15,16,17,18,19,20,21,22], the circuit in [10] uses operational transresistance amplifiers (OTRA), the circuits in [11,12,13] use current-feedback operational amplifiers (CFOA), the circuits in [14,16,17,18] use voltage differencing transconductance amplifiers (VDTA), the circuit in [15] uses voltage differencing differential difference amplifiers (VDDDA), the circuit in [19] uses voltage differencing gain amplifiers (VDGA), while the circuit in [21] uses operational transconductance amplifiers (OTA), and the circuits in [22] use differential difference transconductance amplifiers (DDTA). The shadow filters in [15,16,17,18,19,21,22] offer an electronic tuning capability, but only the filter in [15] offers five filtering functions, namely low-pass (LP), high-pass (HP), band-pass (BP), band-stop (BS), and all-pass (AP). However, the voltage-mode filter in [15] does not provide low-output impedance, which is required for voltage-mode circuits.

This work is focused on the current-mode shadow filter that offers low-input and high-output impedances, which is required for current-mode circuits. With respect to the current-mode shadow filters in [23,24,25,26,27,28,29,30,31], the circuits in [23,24,25,26,27] use current difference transconductance amplifier (CDTA), the circuit in [28] uses operational floating current conveyor (OFCC), the circuit in [29] uses current backwards trans-conductance amplifier (CBTA), while the circuit in [30] uses current controlled current differencing cascadedtransconductance amplifier (CC-CDCTA), and the circuit in [31] uses current conveyor cascaded transconductance amplifier (CCCTA). The shadow filters in [23,24,25,26,27,29,30,31] offer an electronic tuning capability, but only the shadow filter in [31] can offer low-pass, high-pass, band-pass, band-stop, and all-pass filtering functions in one system. The circuit in [31] employs one CCCTA, one EX-CCCTA, and two capacitors. Although the circuit is based on a small number of active blocks, the structure of active blocks is rather complex.

With respect to the mixed-mode shadow filters in [32,33,34], the circuit in [33,34] can realize low-pass, high-pass, band-pass, band-stop, and all-pass filtering functions in the same topology. However, when the mixed-mode circuit in [33] operates in current-mode, the input matching condition, namely *I_in_* = *I_in_*_1_ = *I_in_*_2_, is required. This means that the circuit requires additional circuits to produce multiple copies of a single input signal. The filter in [33] employs two FD-CCCTAs (fully differential current conveyor cascaded transconductance amplifier), three capacitors, and two MOS resistors, while the filter in [34] employs two DDCCCTAs (differential current conveyor cascaded transconductance amplifiers), two capacitors, and one MOS resistor. However, the active block structures used in these filters [34,35] suffer from a relatively high complexity.

This paper presents current-mode shadow filters using CCCIIs with controlled current gain as active elements. The circuits employ three CCCIIs and two grounded capacitors. This work shows that the current gains of the used CCCIIs can perform the role of external amplifiers to adjust the natural frequency and quality factor of the proposed universal filters without the need to modify their internal parameters. The proposed current-mode shadow filters offer low-pass, high-pass, band-pass, band-stop, and all-pass filtering functions in the same topology with low complexity. The natural frequency and the quality factor can be adjusted by the current gains of CCCIIs and can be electronically controlled. The proposed current-mode filters offer low-input and high-output impedances, which is desirable in current-mode circuits. The paper is organized as follows: Section 2 describes the structure of the CCCII with controlled current gain, the proposed current-mode shadow filters and the nonideality analysis. The simulation results of the CCCII with controlled current gain and the shadow filter are shown in Section 3. Section 4 presents the experimental results of the proposed filters and Section 5 concludes the paper.

## 2. Proposed Circuit

### 2.1. CCCII with Controlled Current Gain

**Figure 2:**
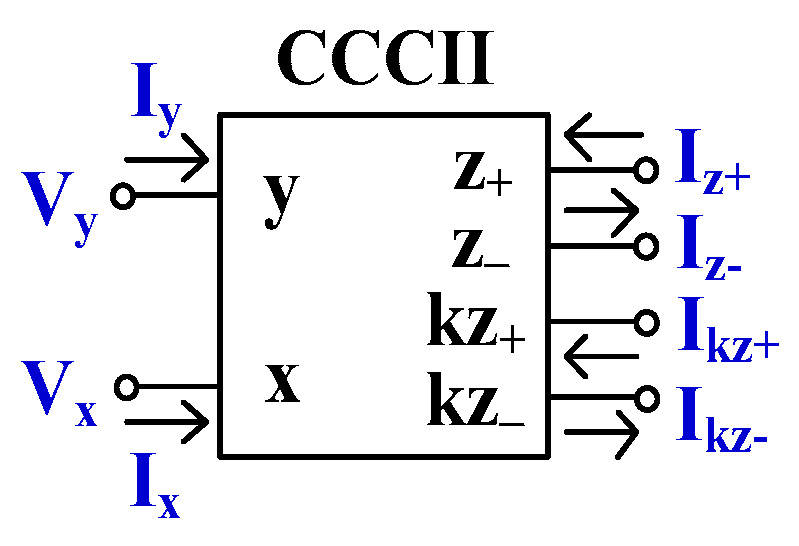
Electrical symbol of the CCCII with controlled current gain.

The electrical symbol of the CCCII with controlled current gain and multiple current outputs is shown in Figure 2. In the ideal case, this element can be described by the following matrix equation:

(2) $$\left(\begin{array}{c} I_{y}\\ V_{x}\\ I_{z\pm }\\ I_{kz\pm } \end{array}\right)=\left(\begin{array}{cccc} 0 & 0 & 0 & 0\\ 1 & R_{x} & 0 & 0\\ 0 & \pm 1 & 0 & 0\\ 0 & \pm k & 0 & 0 \end{array}\right)\left(\begin{array}{c} V_{y}\\ I_{x}\\ V_{z\pm }\\ V_{kz\pm } \end{array}\right)$$

**Figure 3:**
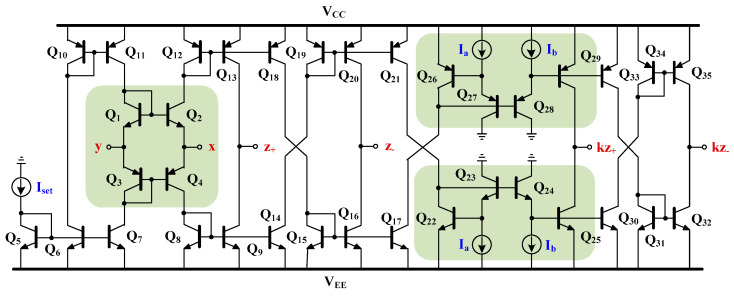
BJT implementation of the CCCII with controlled current gain.

The CCCII with controlled current gain can be implemented using both BJT [5,6,7] as well as CMOS [6,7] technologies. This paper proposes a simple BJT implementation shown in Figure 3. The main circuit consists of the translinear loop (Q_1_-Q_4_) and the positive and negative current mirrors with adjustable gain (Q_22_-Q_25_, Q_26_-Q_29_). Assume that transistors Q_1_ to Q_4_ of a translinear loop are identical and are biased by the current *I_set_*. The parasitic resistance at x-terminal is given by [4]:

(3) $$R_{x}=\frac{V_{T}}{2I_{set}}$$

where *V_T_* is the thermal voltage (~26 mV at 27 °C) and *I_set_* is the bias current. Note that *R_x_* can be controlled by *I_set_*.

Assuming further that transistors Q_22_ to Q_25_ of positive current mirrors and transistors Q_26_ to Q_29_ of the negative current mirrors are identical, the current gain *k* of the CCCII in Figure 3 is given by [5]:

(4) $$k=\frac{I_{a}}{I_{b}}$$

It should be noted that the current gain k can be linearly controlled. Moreover, it is independent of temperature variation.

### 2.2. Proposed Current-Mode Shadow Filter

**Figure 4:**
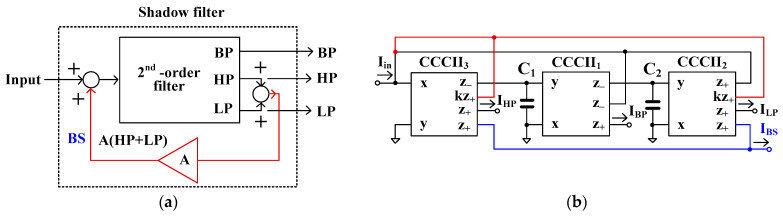
First current-mode shadow filter: (**a**) block diagram, (**b**) first proposed current-mode shadow filter using CCCIIs.

Figure 4a shows the block diagram of the current-mode shadow filter, which consists of a second-order filter (2nd-order filter) that provides three filtering functions i.e., LP, HP, and BP filters, and the amplifier (A) [9]. The outputs of the LP and HP filters are further summed and amplified by the amplifier A, and the output signal of the amplifier is then summed with the input signal. Figure 4b shows the first proposed current-mode shadow filter based on the translinear current conveyors (CCCIIs) with controlled current gains, which is realized based on the block diagram in Figure 4a. The CCCII_1_ to CCCII_3_, C_1_, and C_2_ form a second-order filter that provides three outputs of, LP, HP, and BP filters. This is based on two integrator loops, of which CCCII_1_ and C_1_ create the first integrator, and CCCII_2_, and C_2_ create the second. The current gains of CCCII_2_ (*k*_2_) and CCCII_3_ (*k*_3_) act as an external amplifier (i.e., *k*_2_ = *k*_3_ = *k* = *A*). The outputs of the LP and HP filters are amplified by *k*_2_ and *k*_3_, respectively, and are next fed to the input node of the filter. Thanks to the multiple-output CCCII, the BS filter (*I_BS_*) can be obtained by summing the outputs of the LP and HP filters.

It should be noted that the input current *I_in_* is applied to the x-terminal of CCCII which provides a low impedance level, while the output currents I_LP_, I_HP_, I_BP_, and I_BS_ are supplied from the z-terminals of the CCCII which provides a high impedance level. The circuit uses two grounded capacitors and no passive resistors, which reduces the chip area when it is realized in integrated form.

Using nodal analysis and (2), the output currents of the LP ($I_{LP}$), HP ($I_{HP}$), BP ($I_{BP}$), and BS ($I_{BS}$) filters in Figure 4b can be respectively expressed as

(5) $$I_{LP}=\frac{1}{s^{2}C_{1}C_{2}R_{x1}R_{x2}\left(1+k_{3}\right)+sC_{2}R_{x2}+\left(1+k_{2}\right)}I_{in}$$

(6) $$I_{HP}=\frac{s^{2}C_{1}C_{2}R_{x1}R_{x2}}{s^{2}C_{1}C_{2}R_{x1}R_{x2}\left(1+k_{3}\right)+sC_{2}R_{x2}+\left(1+k_{2}\right)}I_{in}$$

(7) $$I_{BP}=-\frac{sC_{2}R_{x2}}{s^{2}C_{1}C_{2}R_{x1}R_{x2}\left(1+k_{3}\right)+sC_{2}R_{x2}+\left(1+k_{2}\right)}I_{in}$$

(8) $$I_{BS}=\frac{s^{2}C_{1}C_{2}R_{x1}R_{x2}+1}{s^{2}C_{1}C_{2}R_{x1}R_{x2}\left(1+k_{3}\right)+sC_{2}R_{x2}+\left(1+k_{2}\right)}I_{in}$$

By combining the currents $I_{BP}$ and $I_{BS}$, the output current of the AP filter ($I_{AP}$) can be obtained as

(9) $$I_{AP}=\frac{s^{2}C_{1}C_{2}R_{x1}R_{x2}-sC_{2}R_{x2}+1}{s^{2}C_{1}C_{2}R_{x1}R_{x2}\left(1+k_{3}\right)+sC_{2}R_{x2}+\left(1+k_{2}\right)}I_{in}$$

where $R_{x1}$ and $R_{x2}$ are, respectively, the parasitic resistances of CCCII_1_ and CCCII_2_, while $k_{2}$ and $k_{3}$ are, respectively, the current gains of CCCII_2_ and CCCII_3_.

Assuming $k_{2}=k_{3}=k$ (k=A), the natural frequency ($\omega_{o}$) and the quality factor (Q) can be respectively given by

(10) $$\omega_{o}=\frac{1}{\sqrt{C_{1}C_{2}R_{x1}R_{x2}}}$$

(11) $$Q=\left(1+k\right)\sqrt{\frac{C_{1}R_{x1}}{C_{2}R_{x2}}}$$

It should be noted that the parameter $\omega_{o}$ can be electronically controlled by $R_{x1}$ and $R_{x2}$ via $I_{set1}$ and $I_{set2}$ (i.e., $I_{set1}=I_{set2}$) and the parameter Q can be electronically controlled via k (k = $k_{2}$ = $k_{3}$).

From (5), (6), (8), and (9), it can be seen that when the parameter Q is varied by k, the passband gains of the LP, HP, BS, and AP filters change. Namely, increasing the Q value will decrease the passband gains of these filters, except the passband gain of the BP filter, which will be constant.

**Figure 5:**
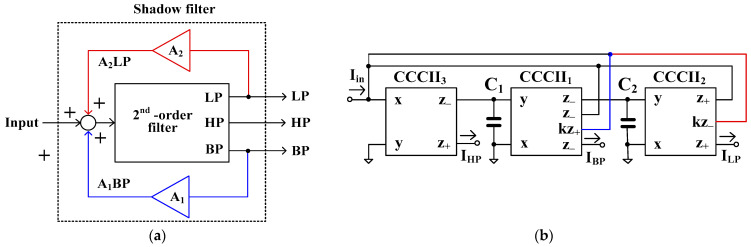
Second current-mode shadow filter: (**a**) block diagram, (**b**) second proposed current-mode shadow filter using CCCIIs.

It should be noted that the shadow filter in Figure 4 uses one external amplifier to modify only the quality factor. Figure 5a shows the block diagram for the second current-mode shadow filter which consists of a 2nd order filter and two amplifiers (A_1_ and A_2_) [9]. The output signals BP and LP are amplified, respectively, by A_1_ and A_2_, and summed with the input signal. Thus, the quality factor and the natural frequency can be independently controlled using the amplifiers A_1_ and A_2_.

Figure 5b shows the proposed second realization of the current-mode shadow filter using CCCIIs with controlled current gains, which realizes the block diagram in Figure 5a. The CCCII_1_ to CCCII_3_, C_1_, and C_2_ form a second-order filter that provides three outputs of the LP, HP, and BP filters, which are similar to the ones in Figure 4b. Compared with Figure 5a, the gain A_1_ is realized by k_1_ of CCCII_1_ and A_2_ is realized by k_2_ of CCCII_2_. The output of the BP filter is amplified by k_1_ and the output of the LP filter is amplified by k_2_. The amplified output signals of the BP and LP filters are applied to the input node of the filter.

Using nodal analysis and (2), the output currents of the LP ($I_{LP}$), HP ($I_{HP}$), BP ($I_{BP}$), and BS ($I_{BS}$) filters of Figure 5b can be respectively expressed as

(12) $$I_{LP}=\frac{1}{s^{2}C_{1}C_{2}R_{x1}R_{x2}+sC_{2}R_{x2}\left(1-k_{1}\right)+\left(1-k_{2}\right)}I_{in}$$

(13) $$I_{HP}=\frac{s^{2}C_{1}C_{2}R_{x1}R_{x2}}{s^{2}C_{1}C_{2}R_{x1}R_{x2}+sC_{2}R_{x2}\left(1-k_{1}\right)+\left(1-k_{2}\right)}I_{in}$$

(14) $$I_{BP}=-\frac{sC_{2}R_{x2}}{s^{2}C_{1}C_{2}R_{x1}R_{x2}+sC_{2}R_{x2}\left(1-k_{1}\right)+\left(1-k_{2}\right)}I_{in}$$

From $I_{LP}$ and $I_{HP}$, the output current of the BS filter ($I_{BS}$) can be obtained as

(15) $$I_{BS}=\frac{s^{2}C_{1}C_{2}R_{x1}R_{x2}+1}{s^{2}C_{1}C_{2}R_{x1}R_{x2}+sC_{2}R_{x2}\left(1-k_{1}\right)+\left(1-k_{2}\right)}I_{in}$$

From $I_{LP}I_{HP}$, and $I_{BP}$, the output current of the AP filter ($I_{AP}$) can be obtained as

(16) $$I_{AP}=\frac{s^{2}C_{1}C_{2}R_{x1}R_{x2}-sC_{2}R_{x2}+1}{s^{2}C_{1}C_{2}R_{x1}R_{x2}+sC_{2}R_{x2}\left(1-k_{1}\right)+\left(1-k_{2}\right)}I_{in}$$

The natural frequency ($\omega_{o}$) and the quality factor (Q) of the filters are given by

(17) $$\omega_{o}=\sqrt{\frac{1-k_{2}}{C_{1}C_{2}R_{x1}R_{x2}}}$$

(18) $$Q=\frac{\sqrt{1-k_{2}}}{1-k_{1}}\sqrt{\frac{C_{1}R_{x1}}{C_{2}R_{x2}}}$$

As can be seen, the parameter $\omega_{o}$ can be controlled by *k*_2_ (*A*_2_) in the range of 0 < *k*_2_ < 1 and the parameter Q can be controlled by *k*_1_ (*A*_1_) in the range of 0 < *k*_1_ < 1. Thus, the parameters $\omega_{o}$ and Q can be independently controlled. It could be noted that adjusting the parameter $\omega_{o}$ by *k*_2_ will affect the parameter Q. In order to provide constant value of the parameter Q, when the parameter$\;\omega_{o}$ is varied by *k*_2_, *k*_1_ must be used to adjust Q.

From (12), (14)–(16), it can be seen that varying the parameter $\omega_{o}$ by k_2_ will affect the passband gains of the LP, BP, BS, and AP filters. Namely, increasing *k*_2_ will increase the passband gains of these filters, except the passband gain of the HP filter, which will be constant.

### 2.3. Impact of Non-Idealities

Taking into account the non-idealities of the CCCII with controlled current gain, its characteristics can be described by the following matrix equation

(19) $$\left(\begin{array}{c} I_{y}\\ V_{x}\\ I_{z\pm }\\ I_{kz\pm } \end{array}\right)=\left(\begin{array}{cccc} 0 & 0 & 0 & 0\\ \alpha & R_{x} & 0 & 0\\ 0 & \pm \beta & 0 & 0\\ 0 & \pm \beta_{k}k & 0 & 0 \end{array}\right)\left(\begin{array}{c} V_{y}\\ I_{x}\\ V_{z\pm }\\ V_{kz\pm } \end{array}\right)$$

where α = 1 − *ε_v_* (with *ε_v_* « 1) denotes the voltage tracking error from y- to x-terminal, β = 1 − *ε_i_* (*ε_i_* « 1) denotes the current tracking error from x- to z-terminals, *β_k_* = 1 − *ε_ik_* (*ε_ik_* « 1) denotes the current tracking error from x- to kz-terminals.

**Figure 6:**
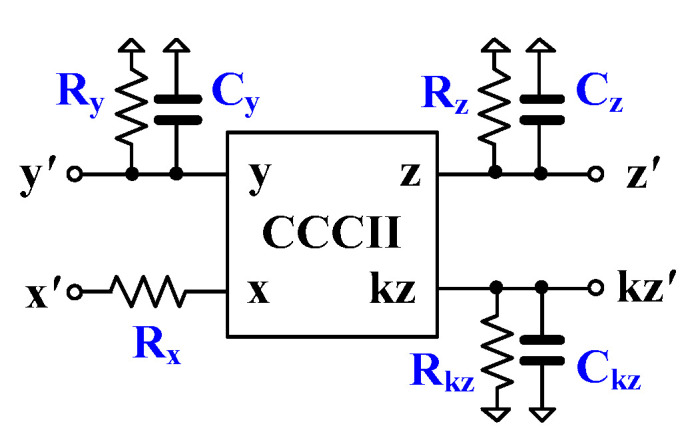
Parasitic resistances and capacitances of CCCII.

The CCCII symbol with non-idealities is shown in Figure 6, where the additional passive elements represent the parasitic resistances and capacitances associated with each terminal of the device. The x-terminal has a parasitic serial resistance R_x_, the y-terminal has a high-value parasitic resistance R_y_ in parallel with a low-value parasitic capacitance C_y_, the z-terminal has a high-value parasitic resistance R_z_ in parallel with a low-value parasitic capacitance C_z_, and the kz-terminal has a high-value parasitic resistance R_kz_ in parallel with a low-value parasitic capacitance C_kz_.

Using (19) and nodal analysis, the denominator (*D*(*s*)) of the transfer functions of the filter in Figure 4b can be expressed by

(20) $$D\left(s\right)=s^{2}C_{1}C_{2}R_{x1}R_{x2}\left(1+k_{3}\beta_{k3}\right)+sC_{2}R_{x2}\alpha_{1}\beta_{1}+\left(\alpha_{1}{\alpha_{2}\beta }_{1}\beta_{2}+k_{2}\beta_{k2}\right)$$

The parameters *ω_o_* and Q in (10) and (11) can be respectively rewritten as

(21) $$\omega_{o}=\frac{1}{\sqrt{C_{1}C_{2}R_{x1}R_{x2}}}\sqrt{\frac{\alpha_{1}{\alpha_{2}\beta }_{1}\beta_{2}+k_{2}\beta_{k2}}{1+k_{3}\beta_{k3}}}$$

(22) $$Q=\frac{1+k_{3}\beta_{k3}}{\alpha_{1}\beta_{1}}\sqrt{\frac{C_{1}R_{x1}}{C_{2}R_{x2}}\cdot \frac{\alpha_{1}{\alpha_{2}\beta }_{1}\beta_{2}+k_{2}\beta_{k2}}{1+k_{3}\beta_{k3}}}$$

Using (19) and nodal analysis, the denominator (*D*(*s*)) of the transfer functions of the filter in Figure 5b can be expressed by

(23) $$D\left(s\right)=s^{2}C_{1}C_{2}R_{x1}R_{x2}+sC_{2}R_{x2}\left({\alpha_{1}\beta }_{1}-k_{1}\beta_{k1}\right)+\left(\alpha_{1}{\alpha_{2}\beta }_{1}\beta_{2}-k_{2}\alpha_{1}\beta_{1}\beta_{k2}\right)$$

The parameters *ω_o_* and Q in (17) and (18) can be respectively rewritten as

(24) $$\omega_{o}=\sqrt{\frac{\alpha_{1}{\alpha_{2}\beta }_{1}\beta_{2}-k_{2}{\alpha_{1}\beta }_{1}\beta_{k2}}{C_{1}C_{2}R_{x1}R_{x2}}}$$

(25) $$Q=\frac{\sqrt{\alpha_{1}{\alpha_{2}\beta }_{1}\beta_{2}-k_{2}\alpha_{1}\beta_{1}\beta_{k2}}}{{\alpha_{1}\beta }_{1}-k_{1}\beta_{k1}}\sqrt{\frac{C_{1}R_{x1}}{C_{2}R_{x2}}}$$

where *α_j_* is the voltage gain error, *β_j_*, and *β_kj_* are the current gain errors of *j*-th CCCII (*j* = 1, 2, 3).

From (24) and (25), it can be seen that the voltage gain errors and the current gain errors of CCCIIs will affect the natural frequency and the quality factor of the proposed shadow filters in Figure 4b and Figure 5b. However, this impact can be compensated by electronic tuning.

Considering the proposed shadow filter in Figure 4b by including CCCII with the parasitic components in Figure 6, the denominator of all filtering functions is given by

(26) $$D\left(s\right)=\left\{s^{2}C_{T1}C_{T2}R_{x1}R_{x2}\left(1+k_{3}\right)+sC_{T2}R_{x2}\left(1+\frac{C_{T1}G_{2}R_{x1}k_{3}+C_{T2}G_{1}R_{x1}k_{3}}{C_{T2}}\right)\;+\left(1+k_{2}\right)\left(1+\frac{G_{2}R_{x1}}{1+k_{2}}\right)\right\}$$

where *C_T_*_1_ = *C*_1_ + *C_z_*_-3_ + *C_y_*_1_, *C_T_*_2_ = C_2_ + *C_z_*_-1_ + *C_y_*_2_, *G*_1_ = (1/*R_z_*_-3_)//(1/*R_y_*_1_), *G*_2_ = (1/*R_z_*_-1_)//(1/*R_y_*_2_).

The parasitic impedance effects can be made negligible by satisfying the following condition:

(27) $$\left.\begin{array}{c} \frac{C_{T1}G_{2}R_{x1}k_{3}\;+\;C_{T2}G_{1}R_{x1}k_{3}}{C_{T2}}\ll 1\\ \frac{G_{2}R_{x1}}{1\;+\;k_{2}}\ll 1 \end{array}\right\}$$

The parasitic capacitance will affect the natural frequency and the quality factor that can be expressed respectively by $\omega_{o}=1/\sqrt{C_{T1}C_{T2}R_{x1}R_{x2}}$ and $Q=\left(1+k\right)\sqrt{C_{T1}R_{x1}/C_{T2}R_{x2}}$, where $k_{2}=k_{3}=k$.

Considering the proposed shadow filters in Figure 5b by including the CCCII with the parasitic components in Figure 6, the denominator of all filtering functions is given by

(28) $$D\left(s\right)=\left\{s^{2}C_{T1}C_{T2}R_{x1}R_{x2}+sC_{T2}R_{x2}\left(\left(1-k_{1}\right)+\frac{C_{T1}G_{2}R_{x1}+C_{T2}G_{1}R_{x1}}{C_{T2}}\right)\;+\left(1-k_{2}\right)\left(1+\frac{G_{1}G_{2}R_{x1}R_{x2}+G_{2}R_{x2}-G_{2}R_{x2}k_{1}}{1-k_{2}}\right)\right\}$$

The parasitic impedance effects can be made negligible by satisfying the following condition:

(29) $$\left.\begin{array}{c} \frac{C_{T1}G_{2}R_{x1}\;+\;C_{T2}G_{1}R_{x1}}{C_{T2}}\ll 1\\ \frac{G_{1}G_{2}R_{x1}R_{x2}\;+\;G_{2}R_{x2}\;-\;G_{2}R_{x2}k_{1}}{1\;-\;k_{2}}\ll 1 \end{array}\right\}$$

The parasitic capacitance will affect the natural frequency and the quality factor that can be expressed, respectively, by $\omega_{o}=\sqrt{\left(1-k_{2}\right)/C_{T1}C_{T2}R_{x1}R_{x2}}$ and $Q=\left(\sqrt{1-k_{2}}/\left(1-k_{1}\right)\right)\sqrt{C_{T1}R_{x1}/C_{T2}R_{x2}}$.

The parasitic impedances of CCCIIs that affect the parameters $\omega_{o}$ and Q of the proposed shadow filters in Figure 4b and Figure 5b can be absorbed by choosing *C*_1_ >> *C_z_*_-3_ + *C_y_*_1_, *C*_2_ >> *C_z_*_-1_ + *C_y_*_2_, *R_x_*_1_ << 1/*G*_1_, *R_x_*_2_ << 1/*G*_2_.

## 3. Simulation Results

The proposed current-mode shadow filters were simulated using SPICE. The CCCII in Figure 2 was designed with the transistor model parameters of AT&T’s ALA400 CBIC-R process [35]. The DC supply voltage was ±2.5 V. The bias currents I_bi_ were fixed to 25 μA and the bias current I_ai_ was used to control the current gain k_i_ (i = 1, 2, 3). The simulated performances of the CCCII with controlled current gain used in this paper are given in [36]. The capacitors C_1_ and C_2_ were 30 nF.

**Figure 7:**
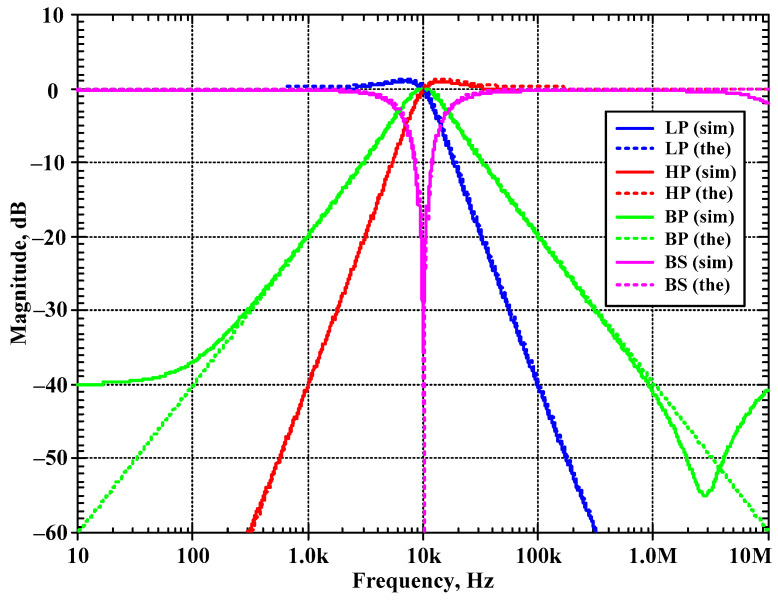
Simulated magnitude frequency responses of the LP, HP, BP, and BS filters of the first current shadow filter in Figure 4b without modification of the natural frequency and the quality factor (sim = simulation, the = theoretical).

**Figure 8:**
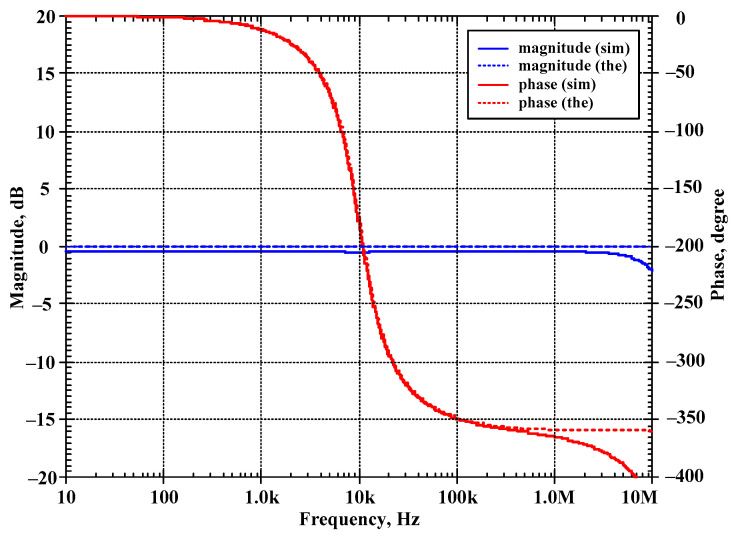
Simulated magnitude and phase frequency responses of the AP filter of the first current-mode shadow filter in Figure 4b without modification of the natural frequency and the quality factor (sim = simulation, the = theoretical).

The first proposed filter in Figure 4b was simulated and the theoretical value was added for comparison. The first simulation was performed with A = 0 (k_2_ = k_3_ = 0), by setting the bias currents I_a2_ = I_a3_ = 0 μA and the bias currents I_set1_ = I_set2_ = I_set3_ = 25 μA. This setting resulted in natural frequency (*f*_o_) of 10.2 kHz and the quality factor (Q) of 1. Figure 7 shows the magnitude frequency responses of the LP, HP, BP, and BS filters and Figure 8 shows the magnitude and phase frequency responses of the AP filters. The simulated natural frequency was 10 kHz and was different from the theoretical value by 1.96%. The bandwidth of the filter was approximately 9 MHz.

**Figure 9:**
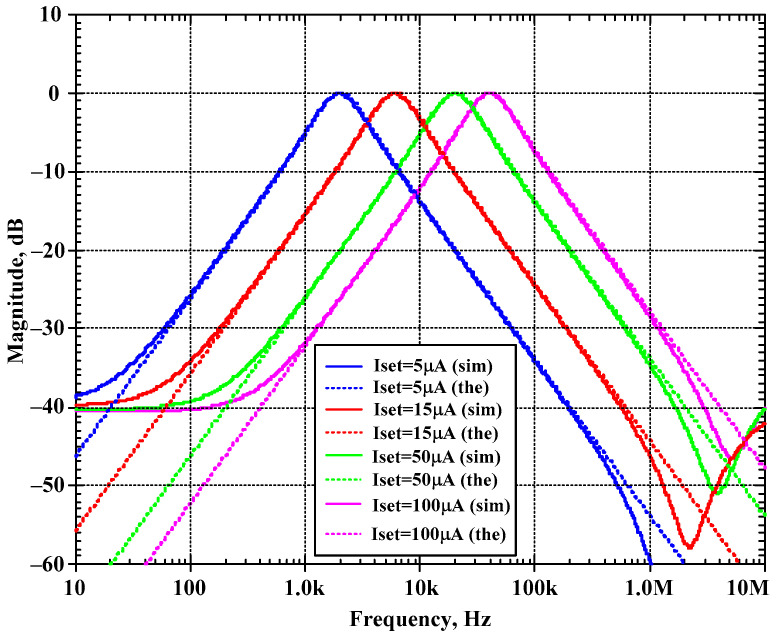
Simulated magnitude frequency responses of the BP filter of the first current-mode shadow filter in Figure 4b with modification of the natural frequency via the bias currents I_set1_ and I_set2_ (I_set1_ = I_set2_ = I_set_) and setting the quality factor (Q) to 1 (Q = 1).

Figure 9 shows the magnitude frequency response of the BP filter for different bias currents I_set1_ and I_set2_ of 5 μA, 15 μA, 50 μA, and 100 μA. The obtained natural frequencies were 1.97 kHz, 5.96 kHz, 19.99 kHz, and 40.22 kHz, respectively. The results of simulations were in close agreement with the theoretical values.

**Figure 10:**
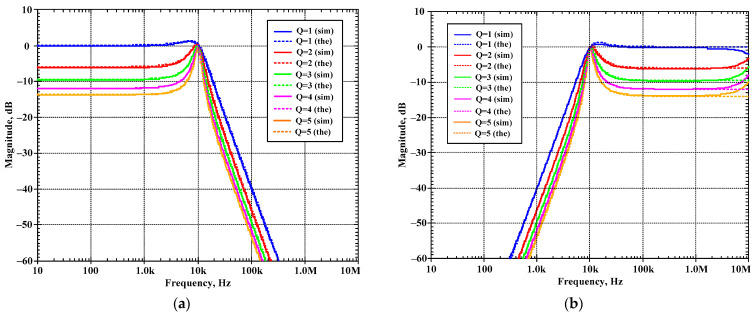

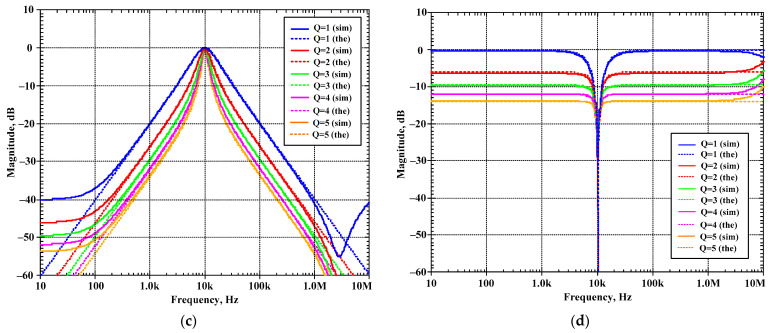
Simulated magnitude frequency responses of the first current-mode shadow filter in Figure 4b, setting the quality factor by the amplifier k for: (**a**) LP, (**b**) HP, (**c**) BP, and (**d**) BS.

**Figure 11:**
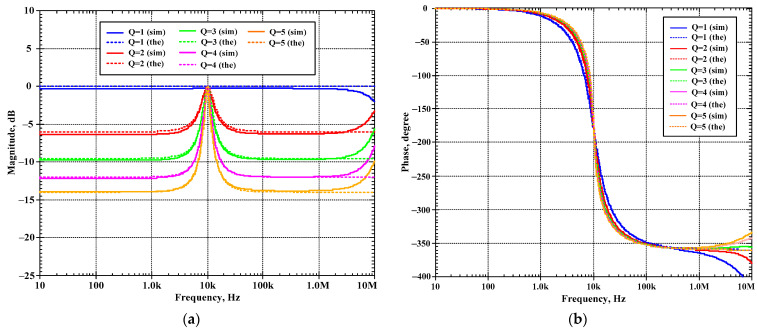
Simulated frequency responses of AP filter of the first current-mode shadow filter in Figure 4b, setting the quality factor by the amplifier k; (**a**) magnitude response and (**b**) phase response.

Figure 10a–d show the magnitude frequency responses of the LP, HP, BP, and BS filters, respectively, when the quality factor (Q) was changed by k (k = k_2_ = k_3_) as Q = 1 (I_a1,2_ = 0 μA), Q = 2 (I_a1,2_ = 25 μA), Q = 3 (I_a1,2_ = 50 μA), Q = 4 (I_a1,2_ = 75 μA), and Q = 5 (I_a1,2_ = 100 μA) while the bias currents I_set1_, I_set2_, and I_set3_ were set to 25 μA. Figure 11a,b show, respectively, the magnitude and phase frequency responses of the AP filter. Figure 10 and Figure 11 are used to confirm that the shadow filter in Figure 4 can modify the quality factor by the gain k (or A).

**Figure 12:**
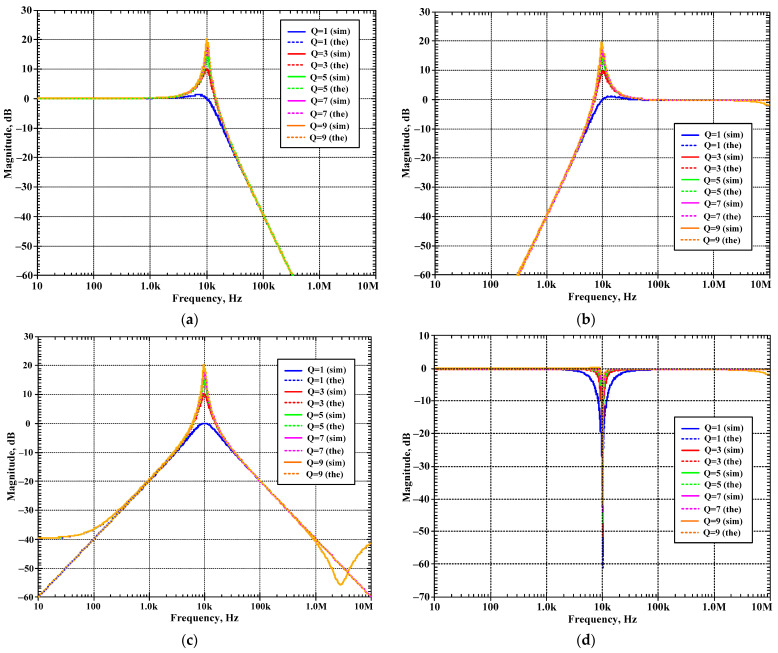
Simulated magnitude frequency responses of the second current-mode shadow filter in Figure 4b, setting the quality factor by the amplifier k_1_ with k_2_ = 0 for: (**a**) LP, (**b**) HP, (**c**) BP, (**d**) BS.

**Figure 13:**
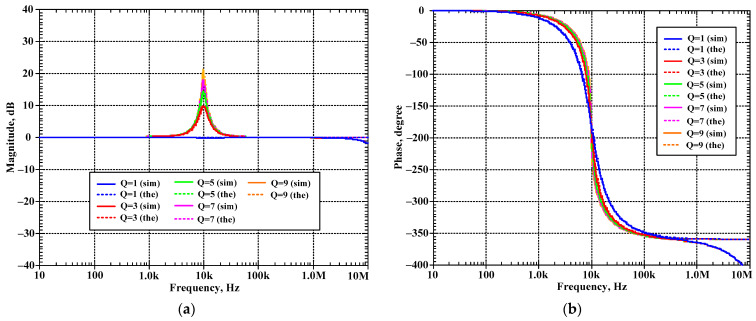
Simulated frequency responses of the AP filter of the second current-mode shadow filter in Figure 4b, setting the quality factor by the amplifier k_1_ with k_2_ = 0; (**a**) magnitude response and (**b**) phase response.

The second proposed current-mode filter in Figure 5b was simulated and compared with the theory. The first simulation was performed with A_2_ = 0 (k_2_ = 0), by setting the bias currents I_a2_ = 0 μA and the bias currents I_set1_ = I_set2_ = I_set3_ = 25 μA while the bias current I_a1_ was used to control the quality factor (Q) as Q = 1 (I_a1_ = 0 μA, k_1_ = 0), Q = 3 (I_a1_ = 16.7 μA, k_1_ = 0.67), Q = 5 (I_a1_ = 20 μA, k_1_ = 0.8), Q = 7 (I_a1_ = 21.5 μA, k_1_ = 0.86), and Q = 9 (I_a1_ = 22.2 μA, k_1_ = 0.88). Figure 12a–d show the magnitude frequency responses of the LP, HP, BP, and BS filters and Figure 13a,b show, respectively, the magnitude and phase frequency responses of AP filter.

**Figure 14:**
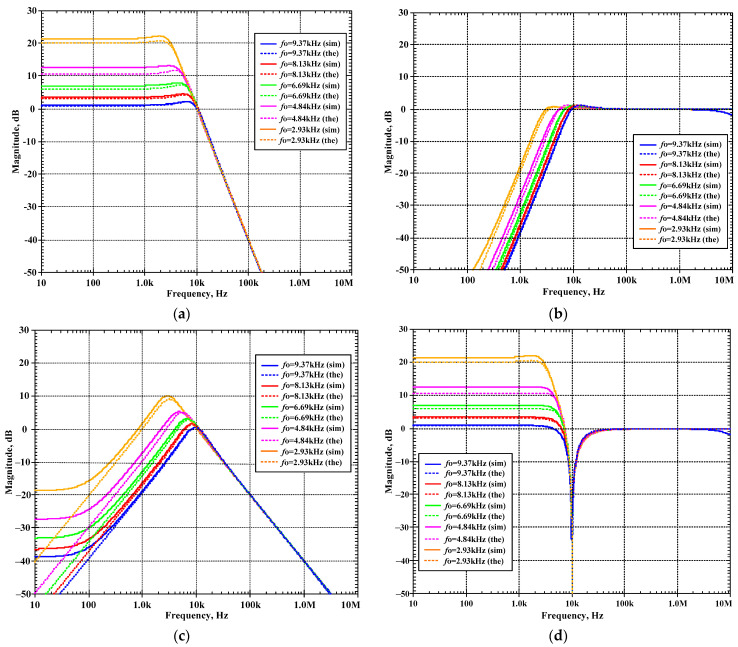
Simulated magnitude frequency responses of the second current-mode shadow filter in Figure 4b, setting the natural frequency by k_2_, while k_1_ is used to adjust Q = 1 for: (**a**) LP, (**b**) HP, (**c**) BP, (**d**) BS.

**Figure 15:**
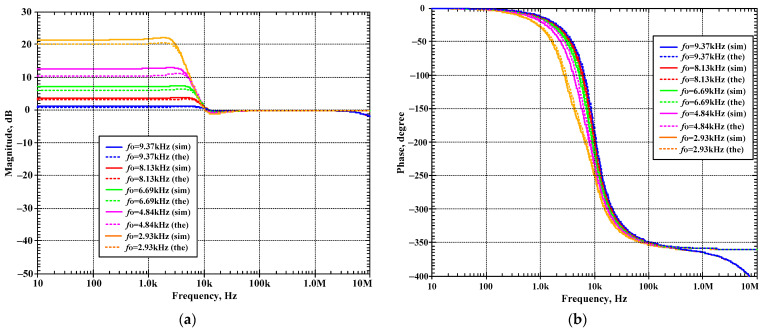
Simulated frequency responses of AP filter of the second current-mode shadow filter in Figure 4b, setting the natural frequency by k_2_, while k_1_ is used to adjust Q = 1; (**a**) magnitude response and (**b**) phase response.

The second proposed shadow filter was simulated when the natural frequency was varied by k_2_ (A_2_) and k_1_ (A_1_) was used for constant Q = 1, and the bias currents I_set1_ = I_set2_ = I_set3_ = 25 μA. Figure 14a–d show the magnitude frequency responses of the LP, HP, BP, and BS filters for *f*_o_ = 9.37 kHz (k_1_ = 0.05 (I_a1_ = 1.25 μA), k_2_ = 0.1 (I_a2_ = 2.5 μA)), *f*_o_ = 8.13 kHz (k_1_ = 0.16 (I_a1_ = 4 μA), k_2_ = 0.3 (I_a2_ = 7.5 μA)), *f*_o_ = 6.69 kHz (k_1_ = 0.29 (I_a1_ = 7.25 μA), k_2_ = 0.5 (I_a2_ = 12.5 μA)), *f*_o_ = 4.84 kHz (k_1_ = 0.45 (I_a1_ = 11.25 μA), k_2_ = 0.7 (I_a2_ = 17.5 μA)), and *f*_o_ = 2.93 kHz (k_1_ = 0.68 (I_a1_ = 17 μA), k_2_ = 0.1 (I_a2_ = 22.5 μA)). Figure 15a,b show, respectively, the magnitude and phase frequency responses of the AP filter.

Figure 12, Figure 13, Figure 14 and Figure 15 are used to confirm that the quality factor and the natural frequency of the shadow filter in Figure 5 can be modified independently by k_1_ (or A_1_) and k_2_ (or A_2_), respectively.

**Figure 16:**
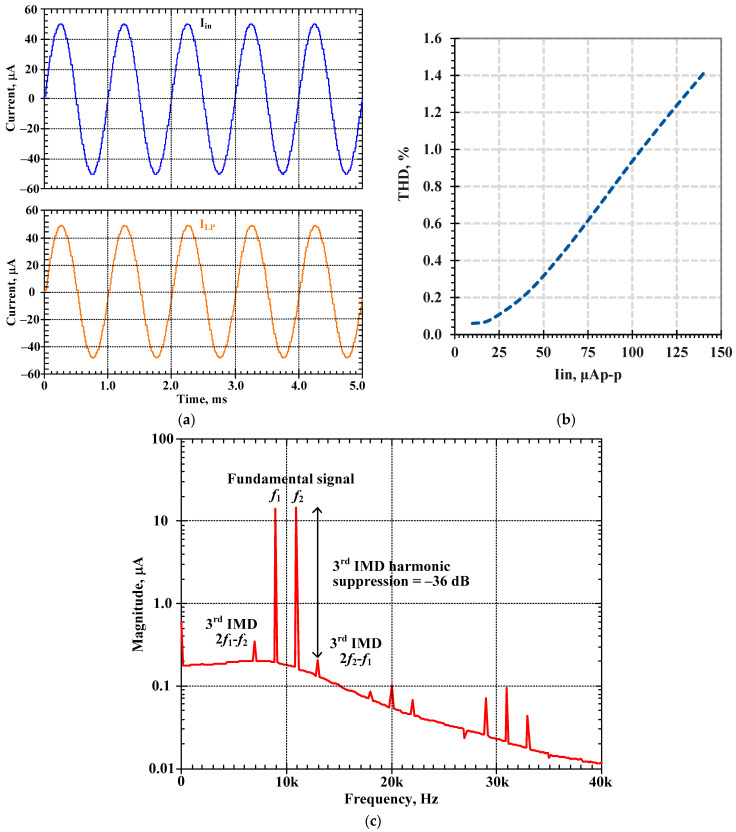
Simulated distortion of first proposed current-mode shadow filter: (**a**) the transient response of the LP filter with THD less than 0.937% for I_in_ = 100 μA_p-p_, (**b**) THD of the LP filter with different amplitude of I_in_, and (**c**) IMD3 of the BP filter.

To investigate the linearity of the proposed shadow filter in Figure 4b, this filter was simulated with Q = 1 (k_2_ = k_3_ = 0, I_a2_ = I_a3_ = 0 μA), where the amplifier A was not active. Figure 16a shows the input and output waveforms of the LP filter for the frequency of 1 kHz, amplitude of 100 μAp-p, and total harmonic distortion (THD) of 0.937%. Figure 16b shows the simulated THD for the frequency of 1 kHz with different input amplitudes. Figure 16c shows the simulated third intermodulation distortion (IMD3) of the BP filter with a two-tone test, with two closely spaced tones f_1_ = 9 kHz and f_2_ = 11 kHz. The IMD3 was less than 1.55% for input amplitude up to 30 μA_p-p_.

**Figure 17:**
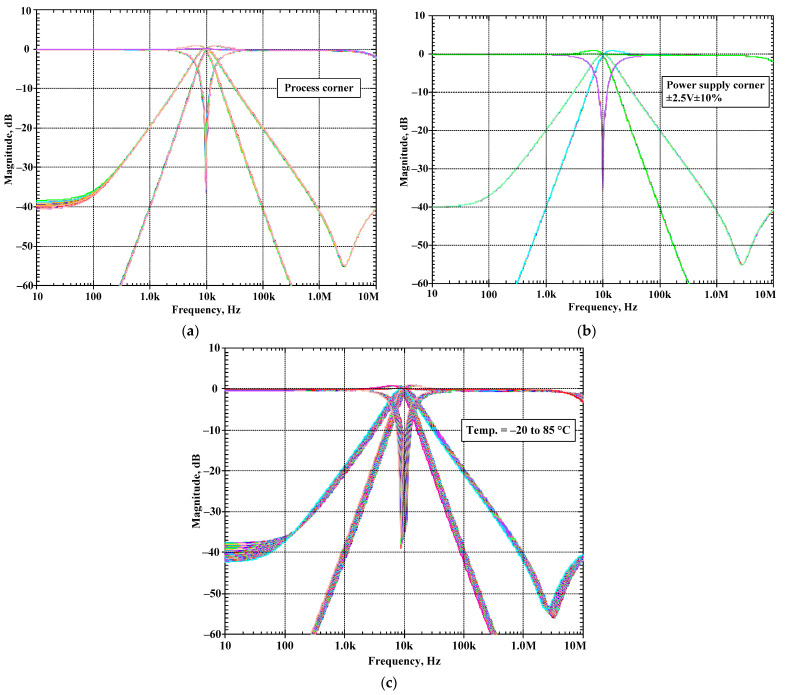
Simulated magnitude responses of the proposed current-mode shadow filter: (**a**) process corner, (**b**) voltage corner, and (**c**) temperature corner.

With the condition of Q = 1 and *f*_c_ = 10 kHz, the proposed current-mode shadow filter in Figure 4b was simulated to investigate the impact of process variations by varying beta (β) in BJT by 10% (LOT tolerance), voltage by varying the supply voltage by ±10%, and temperature (PVT) corners by varying the temperature from −20 to 85 °C. Figure 17a–c show the simulated magnitude frequency responses of the LP, HP, BP, BS, and AP filters for process, voltage, and temperature (PVT) corners, respectively. It can be noted that the natural frequency was affected by the temperature variations. From our investigation, for the temperatures of −20 °C and 85 °C, the natural frequencies were, respectively, 11.09 kHz and 8.81 kHz. Thus, the natural frequency was varied by about ±1.04 kHz.

**Figure 18:**
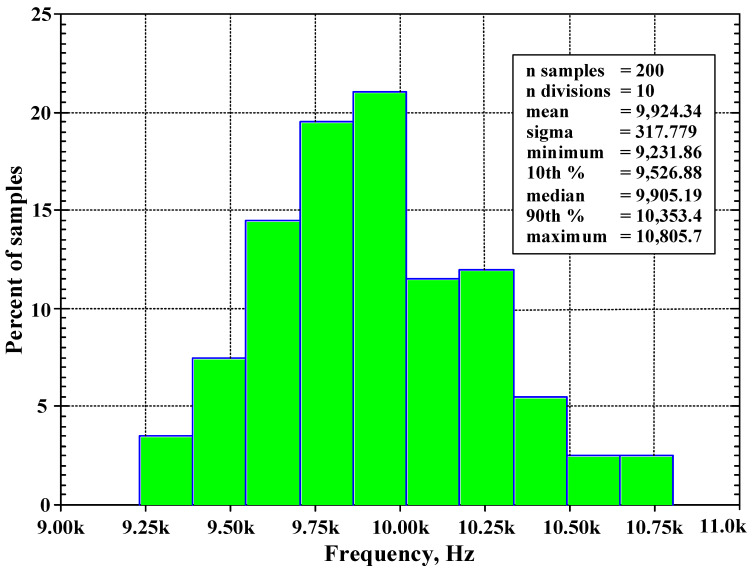
The histogram of the center frequency of the BP filter with 200 runs of MC analysis.

The BP response was simulated by setting 5% tolerances of the capacitor C_1_ and C_2_ at the cut-off frequency of 10 kHz and 200 Gaussian distribution runs. Figure 18 shows the derived histogram of the center frequency. The standard deviation (σ) of *f*_o_ was 0.317 kHz and the maximum and minimum values of *f*_o_ were, respectively, 10.805 kHz and 9.231 kHz.

The proposed current-mode shadow filters were compared with previous shadow filters in Table 1. The voltage-mode shadow filters in [15,22], the current-mode shadow filters in [27,28,30], and the multi-mode shadow filters in [34] were selected for comparison. Compared with [22,27,28], the proposed filter can provide five filtering functions of LP, HP, BP, BS, and AP filters. Compared with [15,30], the proposed filter does not possess a buffer circuit at input or output terminals. Compared with [15,34], the proposed filter has no resistors and has a simpler structure. As can be seen, the proposed filters have the following features which the others do not: they offer the highest number of responses without the need for a buffer circuit at the input and output, have no resistors, all capacitors are grounded, and they have the possibility of electronic tuning of the natural frequency and quality factor.

## 4. Experimental Results

**Figure 19:**
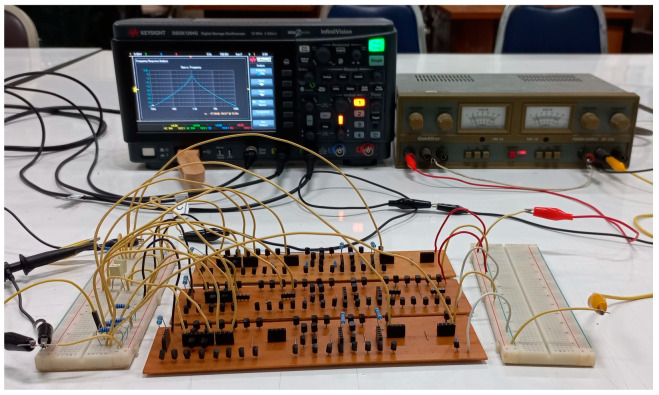
Experimental setup for proposed shadow filters.

To confirm the functionality of the proposed shadow filters, an experimental setup of CCCII was designed using commercially available 2N3904 (NPN) and 2N3906 (PNP) transistors with supply voltage of ±2.5 V. Figure 19 shows the experimental setup for the current-mode shadow filter. Passive capacitors were chosen as C_1_ = C_2_ = 330 nF and passive resistors 10 kΩ were used for voltage-to-current (V-I) converter as input and current-to-voltage (I-V) converter as output. The resistor that was connected in series with terminal I_in_ works as a V-I converter to convert the voltage signal from the function generator to the current signal I_in_ and the grounded resistors that were connected to terminals I_LP_, I_HP_, I_BP_, I_BS_, and I_AP_ were used as I-V converter to convert current signals to voltage signals. The input and output waveforms ware measured using a KEYSIGHT DSOX1204G oscilloscope; the input signal was also provided by this oscilloscope.

**Figure 20:**
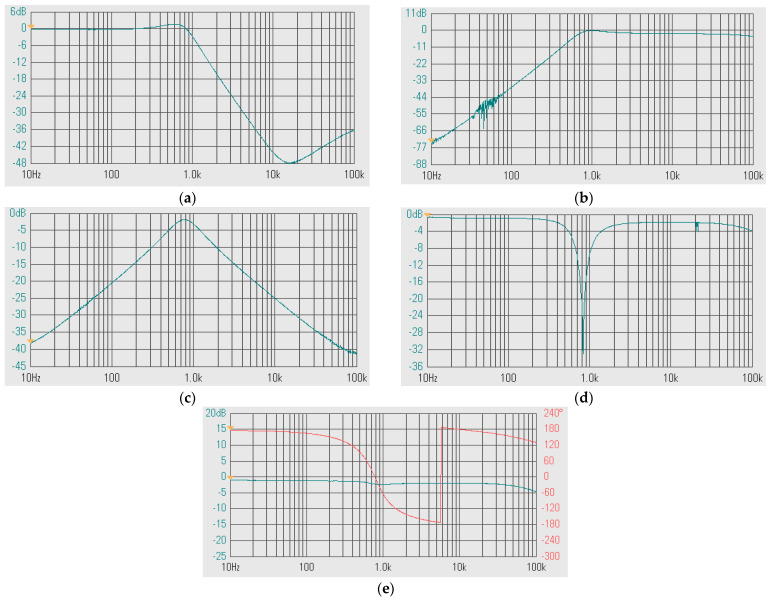
Experimental frequency responses of the first shadow filter in Figure 4b without modification of the natural frequency and the quality factor (A = 0, Q = 1) of (**a**) LP, (**b**) HP, (**c**) BP, (**d**) BS, and (**e**) AP.

**Figure 21:**
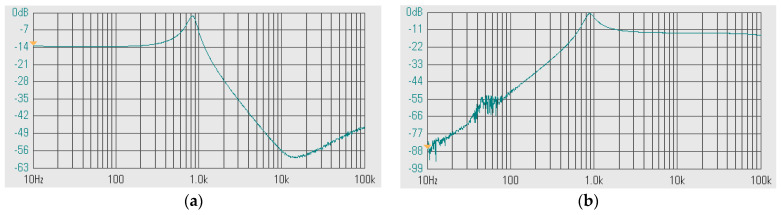

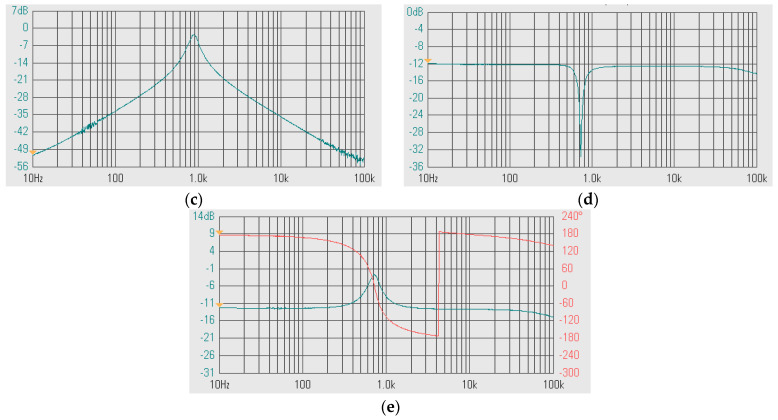
Experimental frequency responses of the first shadow filter in Figure 4b, setting the quality factor by the amplifier A ≈ 3 of (**a**) LP, (**b**) HP, (**c**) BP, (**d**) BS, (**e**) AP.

The experimental frequency responses of the shadow filter in Figure 4b without modification of the natural frequency and the quality factor (A = 0, Q = 1) of (a) LP, (b) HP, (c) BP, (d) BS, and (e) AP are shown in Figure 20. Figure 21 shows the experimental frequency responses of the shadow filter in Figure 4b upon setting the quality factor of the amplifier A ≈ 3 of (a) LP, (b) HP, (c) BP, (d) BS, (e) AP.

**Figure 22:**
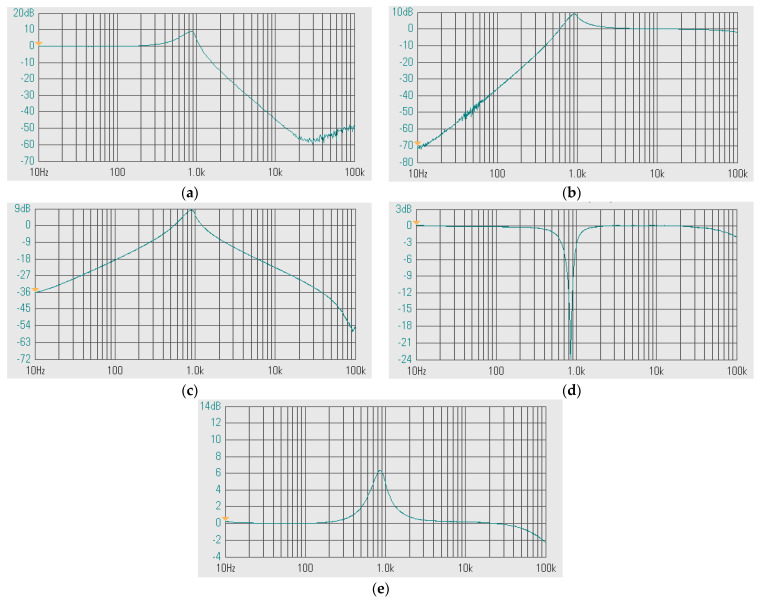
Experimental frequency responses of the second current-mode shadow filter in Figure 5b setting the quality factor by the amplifier k_1_ with k_2_ = 0 for: (**a**) LP, (**b**) HP, (**c**) BP, (**d**) BS, and (**e**) AP.

**Figure 23:**
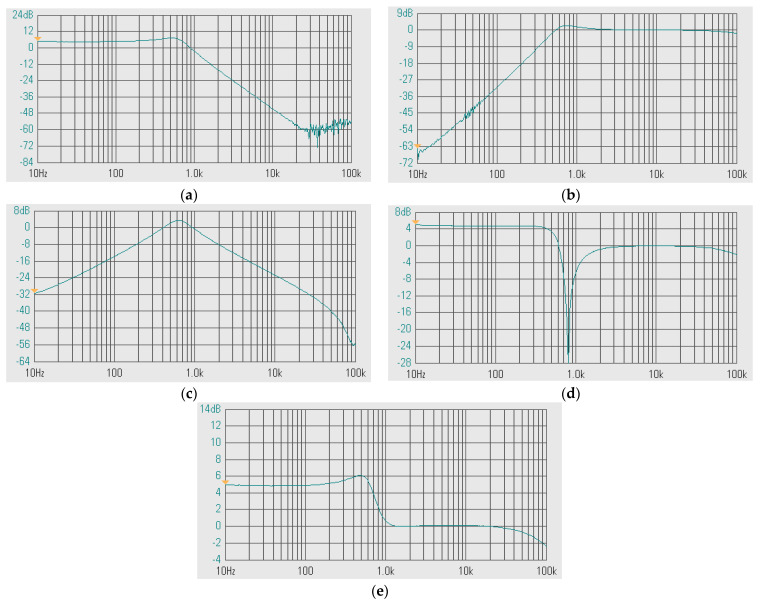
Experimental frequency responses of the second shadow filter in Figure 5b, setting the natural frequency by k_2_, while k_1_ is used to adjust Q for: (**a**) LP, (**b**) HP, (**c**) BP, (**d**) BS, (**e**) AP.

Figure 22 shows the experimental frequency responses of the second shadow filter in Figure 5b, setting the quality factor by the amplifier k_1_ with k_2_ = 0 for: (a) LP, (b) HP, (c) BP, (d) BS. Figure 23 shows the experimental frequency responses of the second shadow filter in Figure 5b, setting the natural frequency by k_2_, while k_1_ is used to adjust Q for (a) LP, (b) HP, (c) BP, (d) BS, (e) AP.

## 5. Conclusions

This paper proposes two current-mode shadow filters using CCCIIs with controlled current gains. The circuits employ three CCCIIs with controlled current gains and two grounded capacitors. The proposed architecture can realize LP, HP, BP, BS, and AP filters in the same topology. The current gains of CCCIIs can be used to modify the natural frequency and the quality factor of all filtering functions. The proposed shadow filters have the advantages of low-input and high-output impedances and low circuit complexity; they do not require passive resistors; they are suitable for integrated circuits by using grounded capacitors; and they have electronic tuning capability. To show the workability and performance of the proposed filters, SPICE simulation was performed. The simulated results were in agreement with the theoretical values and the experimental results confirm the functionality and the performance of the proposed filters.

## Figures and Tables

**Table 1:** Comparison of the proposed design with previous works.

Factor	Proposed	[15]	[22]	[27]	[28]	[30]	[34]
Number of active devices	3 CCCII	3 VDDDA	4 DDTA	7 CDTA	4 OFCC	4 CDCTA, 1 CCII	2 DCCCTA
Realization	BJT process (ALA400 CBIC-R)	0.18 μm CMOS structure & commercial IC	0.18 μm CMOS structure	0.18 μm CMOS structure	0.15 μm CMOS structure & commercial IC	0.18 μm CMOS structure & commercial IC	0.18 μm CMOS structure
Number capacitors	2 C	2C, 1 R	2 C, 3 R	2 C	2 C, 5 R	2 C	2 C, 2 R
Type of filter	SIMO	SIMO	MISO	SIMO	SIMO	MIMO	SIMO
Operation mode	CM	VM	VM	CM	CM	CM	MM
Number of offered responses	5	5	4	1 (BP)	1 (BP)	5	5
No need of buffer circuit at input or output	Yes	No	Yes	Yes	Yes	No	Yes
All grounded capacitors	Yes	Yes	Yes	No	Yes	No	Yes
Electronic control	Yes	Yes	Yes	Yes	Yes	Yes	Yes
Technique to control Q and $\omega_{o}$	CG	EG	EG	EG	EG	EG	EG
Simulated power supply (V)	±2.5	±0.9	0.5	±0.9	±1.5	±1.25	±1.7
Simulated power dissipation (mW)	9.9	-	0.000873	8.53	-	2.23	2.5
Total harmonic distortion (%)	0.937@100μA_pp_	1@280mV_pp_	2@60mV_pp_	<5@100μA_pp_	-	<1@600μA_pp_	<1@200μA_pp_
Verification of result	Sim/Exp	Sim/Exp	Exp	Sim	Sim/Exp	Sim/Exp	Sim/Exp

Note: DCCCTA = differential current conveyor cascaded transconductance amplifier, MM = multi-mode, DDTA = differential difference transconductance amplifier, CG = current gain, EG = external gain (i.e., g_m_R, R_1_/R_2_, and g_m1_/g_m2_).

## Data Availability

Data are contained within the article.
